# Multi-omics study of the anti-colorectal cancer mechanisms of formononetin in *Hedysari Radix*

**DOI:** 10.3389/fphar.2025.1649691

**Published:** 2026-01-06

**Authors:** Jun Rao, Xing Wang, Tanxiu Chen, Mingzi Mo, Conglong Xu, So-Yi Chang, Ssu-Wei Hsu, Xiaoqun Han, Ching-Hsien Chen, Zhi Zheng

**Affiliations:** 1 Jiangxi Cancer Hospital & Institute, The Second Affiliated Hospital of Nanchang Medical College, Jiangxi Clinical Research Center for Cancer, Nanchang, Jiangxi, China; 2 Division of Nephrology, Department of Internal Medicine, University of California, Davis, Davis, CA, United States; 3 Comprehensive Cancer Center, University of California, Davis, Sacramento, CA, United States; 4 Jiangxi Provincial People’s Hospital, The First Affiliated Hospital of Nanchang Medical College, Nanchang, Jiangxi, China; 5 Institute of Neurology and Department of Neurology, Jiangxi Academy of Clinical Medical Sciences, The First Affiliated Hospital, Jiangxi Medical College, Nanchang University, Nanchang, Jiangxi, China; 6 Yichun University, Yichun, China; 7 Jiangxi Jingde Traditional Chinese Medicine Co., Ltd., Jingdezhen, Jiangxi, China

**Keywords:** anti-tumor, CA9, colorectal cancer, formononetin, hedysari radix, MME

## Abstract

**Background:**

*Hedysari Radix* (HR), commonly known as *Hong-Qi* in Chinese, is a traditional Chinese herbal medicine recognized for possessing anti-inflammatory and anti-tumor properties. While the polysaccharides in HR have been extensively studied, other HR metabolites and their potential anti-tumor properties remain largely unknown.

**Methods:**

We employed a multi-omics strategy integrating metabolomics, network analysis, proteomics, phosphoproteomics, and molecular docking to identify HR metabolites with anti-colorectal cancer (CRC) property and investigate underlying mechanisms.

**Results:**

Using mass spectrometry-based metabolomics, we identified 1,292 metabolites across eight processed HR products. Key metabolites including medicarpin, formononetin, naringenin, and quercetin were validated via the Traditional Chinese Medicine Systems Pharmacology Database and Analysis Platform (TCMSP). Notably, formononetin-derived metabolites were significantly enriched during HR processing. The metabolite-metabolite correlation analysis revealed key compounds such as flavonoids and formononetin. Subsequent network analysis combined with label-free data-independent acquisition (DIA) proteomics and phosphoproteomics in colon cancer cells identified 194 potential targets, 291 differentially expressed proteins, and 1,535 phosphorylated proteins that were regulated by formononetin. Cell-surface enzymes carbonic anhydrase IX (CA9) and membrane metalloendopeptidase (MME) were consistently identified in different analyses as key targets, and molecular docking results confirmed their strong binding to formononetin. Bioinformatics analyses further revealed significant enrichment of cancer-associated pathways, including PI3K-Akt, Hippo, HIF-1 signaling, and cholesterol metabolism upon formononetin treatment.

**Conclusion:**

The findings provide novel insights into the HR metabolome and reveal the multi-targeting roles of formononetin in CRC development, laying the foundation for developing new CRC therapeutic strategies.

## Graphical Abstract

**Figure:**
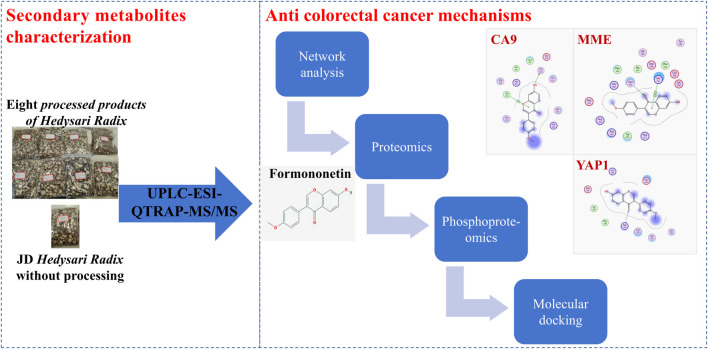

## 1 Introduction

Colorectal cancer (CRC) is the third most common malignancy and the second leading cause of cancer-related deaths worldwide (Morgan et al., 2023). Global CRC incidence is projected to reach 3.2 million by 2040, posing a significant public health challenge. Current CRC treatments include surgery, radiotherapy, chemotherapy, targeted therapy, and immunotherapy. Patients with stage I-III CRC typically undergo radical surgery, and patients with metastatic stage IV CRC receive systemic therapies including conventional chemotherapeutic agents (e.g., 5-Fluorouracil, Irinotecan, Oxaliplatin), targeted therapeutic drugs (e.g., Aflibercept, Bevacizumab, Cetuximab, Panitumumab), and immune checkpoint inhibitors (e.g., Pembrolizumab, Nivolumab) (El Bali et al., 2021; Groelly et al., 2023; Miyamoto et al., 2023). However, these treatments often face problems such as immune suppression, chemotherapy resistance, toxic side effects, and high recurrence rates, making it necessary to develop more effective and less toxic alternatives.

Traditional Chinese medicine (TCM) has been used for thousands of years in China and other Asian countries to treat various diseases (Huang et al., 2024). As a complementary therapy for cancers including CRC, lung cancer, breast cancer, and ovarian cancer, TCM has been demonstrated to be able to enhance chemotherapy efficacy while reducing side effects and toxicity (Wan et al., 2022; Wei et al., 2023; Wang et al., 2024; Yu et al., 2024). TCM has shown benefits in regulating immune responses and protecting colonic mucosal barrier in stage II and III CRC patients, making it a potential long-term treatment strategy for late stage CRC (Feng et al., 2021; Wang et al., 2021; Wan et al., 2022). *Hedysari Radix* (*Hedysarum polybotrys* Hand.-Mazz. [Fabaceae], or HR), a well-known TCM, is widely used due to its diverse antioxidant, immunomodulatory, anti-inflammatory, and anti-tumor effects (Mo et al., 2022). While HR’s anti-tumor effects in lung, gastric, liver, and ovarian cancers are mostly credited to its polysaccharides, other bioactive compounds in HR like terpenoids and flavonoids also demonstrated significant anti-tumor properties and attracted broad research interests.

Advances in “omics” technologies, such as metabolomics and proteomics, have significantly contributed to deeper understanding of therapeutic mechanisms in many diseases including cancer (Zhu et al., 2022). Among them, metabolomics has been instrumental in identifying small molecule bioactive ingredients in TCM to elucidate their effects (Wang et al., 2017). In an integrated metabolomics and network pharmacology analysis (Qu et al., 2022), it was revealed that dandelion could suppress tumor progression in triple-negative breast cancer (TNBC) by modulating cell cycle signaling and metabolic pathways. Studies using proteomics and phosphoproteomics have focused on identifying molecular targets of TCMs to facilitate the development of novel cancer therapeutic agents (Wang et al., 2019; Song et al., 2022; Xia et al., 2022). One TCM ingredient, triptolide, has been demonstrated to be able to suppress CRC proliferation by regulating key proteins and pathways including PI3K/AKT and Hedgehog signaling (Song et al., 2022).

In this study, we integrated metabolomics, network analysis, proteomics, and phosphoproteomics to investigate the anti-tumor mechanisms of HR. We investigated eight heat-processed HR products and SW620 colon cancer cells to identify potentially key active metabolites and their molecular targets. Additionally, we analyzed metabolic changes during the heat-processing (called *paozhi in Chinese*) of HR to elucidate the therapeutic effects generated from the processing against CRC.

## 2 Materials and methods

### 2.1 Sample collection

Eight heat-processed HR products (labeled JZ, JPT, XFT, TQT, JJ, SFT, MDYY, and JD) were purchased from different manufacturers in China. Unprocessed JD HR was also obtained and designated SJD HR. Each HR product was prepared in triplicate, air-dried, flash-frozen in liquid nitrogen, and stored at −80 °C until analysis.

### 2.2 Metabolomics analysis

Targeted metabolomics analysis focusing on secondary metabolites was conducted following the standards outlined in the ConPhyMP statement (Supplementary Tables S1, S2) (Heinrich et al., 2022; Wang et al., 2022; Sun et al., 2023). Metabolites were extracted with 1,200 μL pre-cooled (−20 °C) 70% aqueous methanol containing 2-chlorophenylalanine (1 ppm; purity 98%) as an internal standard. After vortexing and centrifugation, the supernatant was filtered through a 0.22 μm microporous membrane (ANPEL, Shanghai, China) and transferred to an injection vial. Metabolomics analysis was performed using an ultra-high-performance liquid chromatography system (Nexera X2 UPLC, Shimadzu, Tokyo, Japan) coupled with an electrospray ionization-triple quadrupole-linear ion trap mass spectrometer (4500 QTRAP mass spectrometer, Applied Biosystems, Waltham, MA, USA). Chromatographic separation was achieved using an Agilent SB-C18 column (1.8 µm, 2.1 mm × 100 mm) with mobile solution A (0.1% formic acid in water) and solution B (0.1% formic acid in acetonitrile). Gradient elution was performed at a flow rate of 0.35 mL/min with an injection volume of 2 μL. The gradient program was: 95% A/5% B at 0 min, linearly ramped to 5% A/95% B at 9.0 min and held until 10.0 min, then returned to 95% A/5% B at 11.1 min and maintained until 14.0 min. The elution was directly introduced into the mass spectrometer with key operational parameters as follows: turbo spray ion source, source temperature 550 °C, ion spray voltage 5500 V (positive) and 4500 V (negative), ion source gas I 50 psi, gas II 60 psi, curtain gas 25 psi (Wang et al., 2022; Sun et al., 2023). The declustering potential and collision energy were individually optimized for each transition. Metabolite identification and quantification were conducted by searching the Metware Database (MWDB, MetWare Biological Co., Ltd., Wuhan, China). Chromatographic peaks were integrated and corrected using MultiQuant software (AB SCIEX, Framingham, MA, USA). SIMCA-P 18.0 software was used for principal component analysis (PCA), while significantly changed metabolites were determined using the orthogonal partial least squares discriminant analysis (OPLS-DA), followed by an independent t-test (SPSS 22.0 software) (Wan et al., 2022). VIP (Variable Importance in Projection) values from the OPLS-DA model reflected the contribution of each variable, with VIP values >1 indicating significance. The natural variation of the content of each secondary metabolite in HR was presented in a Z-score plot generated using R software (version 4.0.2) with three SD as the cutoff. Metabolite-metabolite correlation analysis was also performed using R software for Pearson’s product-moment correlation (Pearson’s r), p-values were calculated using the cor.test function.

### 2.3 Network analysis for target selection

The Simplified Molecular Input Line Entry System (SMILES) structure of formononetin was retrieved from PubChem (https://pubchem.ncbi.nlm.nih.gov/) to predict its potential molecular targets based on its structural properties and bioactivity profiles. Target prediction was performed in SwissTargetPrediction (http://www.swisstargetprediction.ch/), TargetNet (http://targetnet.scbdd.com/), and PharmMapper (http://lilab-ecust.cn/pharmmapper/submitfile.html), which integrate multiple computational approaches to achieve comprehensive coverage of plausible targets. Duplicate entries were removed to retain unique targets. Targets related to CRC were compiled by querying DisGeNET (https://www.disgenet.org/), GeneCards (https://www.genecards.org/), and OMIM (https://www.omim.org/) databases using the keyword “Colorectal cancer” to select genes with documented relevance to CRC pathogenesis. Middle targets between formononetin and CRC were identified using Venny 2.1.0 (https://bioinfogp.cnb.csic.es/tools/venny/) to generate a Venn diagram including potential therapeutic targets of formononetin in CRC. The overlapped targets were then selected and subjected to Gene Ontology (GO) and Kyoto Encyclopedia of Genes and Genomes (KEGG) pathway enrichment analyses via the Bioinformatics online platform (https://www.bioinformatics.com.cn/) to elucidate the biological processes and signaling pathways involved.

### 2.4 Cell culture and cell counting kit-8 (CCK-8) assay

Human SW620 colon cancer cells (ATCC, Manassas, VA) were cultured in RPMI-1640 medium supplemented with 10% fetal bovine serum at 37 °C in a 5% CO_2_ incubator. Cells were seeded in 96-well plates (2,000 cells/well) and treated with formononetin (catalog #F408902, Aladdin, Shanghai, China) at final concentrations of 0, 5, 10, 20, 40, and 80 μM for 48 h. Cell viability was assessed using CCK-8 reagent (catalog #DB884, Dojindo, Kumamoto, Japan). After 1 hour of incubation with CCK-8, absorbance at 450 nm was measured using a microplate spectrophotometer.

### 2.5 Proteomic analysis

Proteomic profiling was performed using a label-free DIA approach to identify protein changes in formononetin-treated SW620 cells as previously reported (Wang et al., 2020; Wan et al., 2022). Proteins were extracted, reduced, and digested using the Filter-Aided Sample Preparation (FASP) method and analyzed on an EASY-nLC 1,200 system (Thermo Fisher Scientific, San Jose, CA, USA) coupled to a Q Exactive HF mass spectrometer (Thermo Fisher Scientific, San Jose, CA, USA). Chromatographic separation was performed on a reversed-phase C18 column with a binary gradient mobile solutions comprising 0.1% formic acid in water (A) and 0.1% formic acid in 80% acetonitrile (B). The gradient program was: 6%–10% B (0–5 min), 10%–30% B (5–47 min), 30%–45% B (47–55 min), and 95% B (55–60 min). MS/MS analysis in DIA mode was set at a resolution of 30,000, 32 isolation windows (first mass: 200 m/z), automatic maximum injection time, an AGC target of 3e6, and normalized collision energy of 28% (Wang et al., 2020). DIA raw data was processed using Spectronaut 18.0 (Biognosys) for protein identification. Differentially expressed proteins were defined by a fold change >1.2 and *p* value <0.05. GO and KEGG enrichment analyses of these proteins were conducted using OmicsBean (http://www.omicsbean.cn). The raw MS data have been deposited to the ProteomeXchange Consortium (https://proteomecentral.proteomexchange.org) via the iProX partner repository with the dataset identifier PXD059226 (Chen et al., 2022).

### 2.6 Phosphoproteomic analysis

Phosphoproteomic analysis was performed on formononetin-treated SW620 cells using DIA-based methods. Protein extraction, digestion, and phosphopeptide enrichment were carried out using the High-Select™ Fe-NTA Kit (Thermo Fisher Scientific, San Jose, CA, USA) following the manufacturer’s instructions (Song et al., 2022). Briefly, lyophilized peptides were reconstituted in 200 μL of immobilized metal affinity chromatography (IMAC) binding/wash buffer. IMAC spin columns were activated by centrifugation (1,000 × g, 30 s) and equilibrated with two washes of the same buffer. After loading the samples, the resin was gently resuspended and incubated for 30 min with manual mixing at 10-min intervals. Following incubation, the columns were washed three times with binding/wash buffer and once with HPLC-grade water. Phosphopeptides were eluted twice with 100 μL elution buffer each time (1,000 × g, 30 s each), and the two elution were combined and dried under vacuum. The eluted phosphopeptides were analyzed on an EASY-nLC 1,200 system coupled to a Q Exactive HF mass spectrometer under the same parameters as in the proteomic analysis. Phosphorylation site abundances were normalized by dividing the abundance of each site by its corresponding protein abundance (Fan et al., 2020). Phosphorylation sites exhibiting significant alterations (fold change >1.2 and *p* < 0.05) were subjected to functional annotation through GO and KEGG pathway enrichment analyses.

### 2.7 Molecular docking

Molecular docking simulations were conducted using MOE software (version 2019.0102). The molecular structures of formononetin and four reference drugs (5-Fluorouracil, Capecitabine, Oxaliplatin, and Irinotecan) were retrieved from the PubChem database. The structures of target proteins CA9, MME, and YAP1 were obtained from the Protein Data Bank (https://www.rcsb.org/). Protein structures were processed in MOE, and semi-flexible docking simulations were performed to evaluate interactions between formononetin and the selected targets.

## 3 Results

### 3.1 Metabolite profiling of HR

**FIGURE 1:**
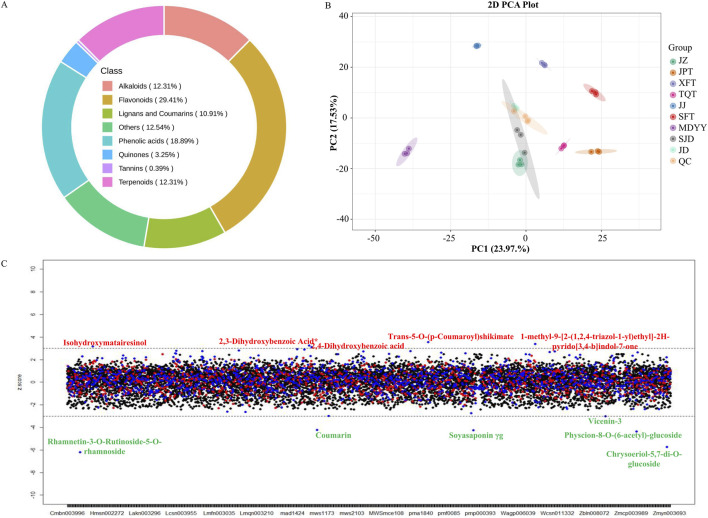
**(A)** Classification of the 1,292 detected metabolites in HR according to UPLC-ESI-QTRAP-MS/MS analysis. **(B)** PCA score plot generated from the metabolomic data across the detected samples. **(C)** Z-score plot of the 1,292 detected metabolites in the HR samples. Metabolites labeled in red or green represent those that are increased or decreased, respectively, in SJD HR.

A targeted UPLC-ESI-QTRAP-MS/MS metabolomic analysis focusing on secondary metabolites was conducted on eight heat-processed HR products from different manufacturers. We identified 1,292 metabolites (Supplementary Table S3), classified into eight major categories: flavonoids, phenolic acids, terpenoids, alkaloids, lignans and coumarins, and others. Flavonoids constituted the largest category (29.41%), followed by phenolic acids (18.89%), others (12.54%), alkaloids (12.31%), terpenoids (12.31%), and lignans and coumarins (10.91%) (Figure 1A). There were 26 metabolites that accounted for more than 1% each of the total relative content of the HR metabolome, such as 3-hydroxy-1-methylpyrrolidin-2-one, hypaphorine, formononetin, and ferulic acid. Notably, formononetin and its five derivatives including formononetin-7-O-glucoside and formononetin-7-O-(6″-malonyl)glucoside constituted approximately 4.22% of the total HR metabolome, while the most abundant single metabolite, 3-hydroxy-1-methylpyrrolidin-2-one, was 3.06% of the HR metabolome.

We further searched the Traditional Chinese Medicine Systems Pharmacology Database and Analysis Platform (TCMSP) to screen for metabolites with potential drug activities (Li et al., 2023). The selection criteria were oral bioavailability (OB) ≥30%, drug-likeness (DL) ≥0.18, molecular weight (MW) ≤500, logP ≤5, hydrogen bond donors (nOHNH) ≤5, and hydrogen bond acceptors (nOH) ≤10. The criteria were consistent with standard TCMSP thresholds. OB ≥ 30% ensures sufficient systemic exposure after oral administration, DL cutoff of 0.18 corresponds to the mean value of known drug molecules in DrugBank. Using these criteria, we identified 11 metabolites with potential drug activity (Table 1), and six of them—medicarpin, calycosin, formononetin, naringenin, quercetin, and vestitol—were detected in our metabolomic profiling of HR.

**TABLE 1:** Predicted metabolites with potential activity in HR satisfying OB ≥ 30%, DL ≥ 0.18, MW ≤ 500, miLogP ≤ 5, nOHNH ≤ 5, and nOH ≤ 10.

Mol ID	Molecule name	MW	AlogP	Hdon	Hacc	OB (%)	DL	The relative content measured in metabolomic analysis
MOL011076	(+)-Medicarpin	270.3	2.66	1	4	60.46	0.34	0.74%
MOL004941	(2R)-7-hydroxy-2-(4-hydroxyphenyl)chroman-4-one	256.27	2.57	2	4	71.12	0.18	Not detected
MOL011078	3′,7-dihydroxy-4′-methoxy-isoflavone	286.3	2.43	2	5	50.7	0.24	Not detected
MOL000417	Calycosin	284.28	2.32	2	5	47.75	0.24	0.25%
MOL001792	4′,7-Dihydroxyflavanone	256.27	2.57	2	4	32.76	0.18	Not detected
MOL000392	formononetin	268.28	2.58	1	4	69.67	0.21	1.46%
MOL005575	Gentiacaulein	288.27	2.4	2	6	72.82	0.27	Not detected
MOL004328	Naringenin	272.27	2.3	3	5	59.29	0.21	0.05%
MOL000098	Quercetin	302.25	1.5	5	7	46.43	0.28	0.01%
MOL004644	Sainfuran	286.3	3.38	2	5	79.91	0.23	Not detected
MOL000500	Vestitol	272.32	3.15	2	4	74.66	0.21	0.24%

### 3.2 Metabolic changes during heat processing of HR

To examine metabolic alterations induced by heat-processing (called *paozhi* in Chinese) of HR, we performed UPLC-ESI-QTRAP-MS/MS analysis on SJD, the unprocessed form of JD HR. The PCA plot demonstrated clear separation between the three replicates of SJD HR and the other heat-processed HR samples including heat processed JD HR (Figure 1B). Processed HR samples clustered more tightly than SJD HR, indicating increased metabolic consistency post-processing. Z-score analysis identified 11 metabolites in SJD HR as outliers relative to processed HR products, including rhamnetin-3-O-rutinoside-5-O-rhamnoside, chrysoeriol-5,7-di-O-glucoside, physcion-8-O-(6-acetyl)-glucoside, and trans-5-O-(p-coumaroyl)shikimate (Figure 1C). Six metabolites had increased levels post-processing, and three of them were flavonoids, including Rhamnetin-3-O-rutinoside-5-O-rhamnoside and chrysoeriol-5,7-di-O-glucoside whose absolute Z-scores were bigger than 5.

**FIGURE 2:**
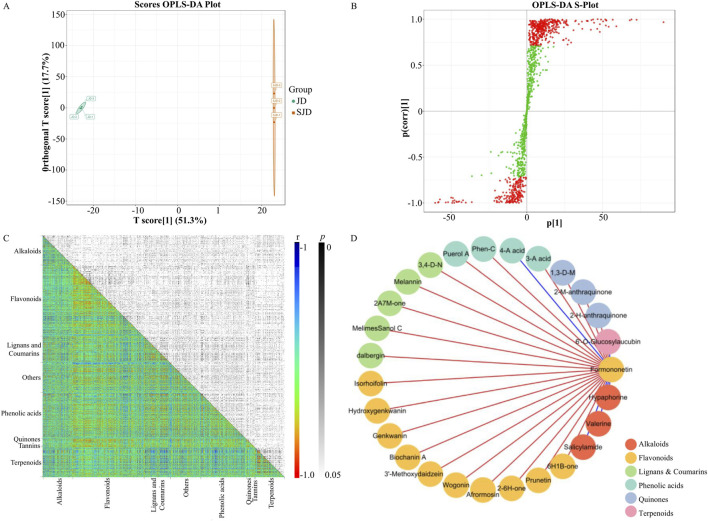
**(A)** Score plot from the OPLS-DA model generated from the metabolomic data of JD HR and its original material, SJD HR. **(B)** S-plot from the OPLS-DA model generated from the metabolomic data of JD HR and its original material, SJD HR. Metabolites with a VIP value greater than one in the OPLS-DA model are labeled in red, while metabolites with a VIP value less than one are labeled in green. **(C)** Metabolites-metabolites correlation/significance in HR. X and Y-axes were categorized into metabolites. Both r and *p* values of the correlations were shown in distinct colors. **(D)** The regulatory metabolic network based on formononetin-related significant correlations (r^2^ ≥ 0.81; *p* ≤ 0.05). Metabolites were represented as nodes, displayed different colors in different categories. The relations among metbolites were represented as edges. The positive correlations were displayed in red while the negative ones were displayed in blue. 6-hydroxy-2-(3-methoxybenzylidene)-1-benzofuran-3(2H)-one*: 6H1B-one; 2-(3,4-Dimethoxybenzylidene)-6-Hydroxy-1-Benzofuran-3(2H)-One*: 2–6H1B-one; 2-acetyl-7-methoxy-3H-benzo [f]chromen-3-one: 2A7M-one; 3,4-Dihydro-4-(4-hydroxy-3-methoxyphenyl)-3-(hydroxymethyl)-6,7-dimethoxy-(3R,4S)-2-naphthalenecarboxaldehyde: 3,4-D-N; 4-Aminobenzoic acid: 4-A acid; 3-Aminosalicylic acid: 3-A acid; 1,3-dihydroxy-6-methoxy-7-methylanthraquinone: 1,3-D-M; 2-Methyl-1,3,6-trihydroxy-9,10-anthraquinone: 2-M-anthraquinone; 2-hydroxy-3-hydroxymethyl-anthraquinone: 2-H-anthraquinone.

The metabolic differences between the unprocessed and processed JD HR were also investigated using supervised OPLS-DA, and 108 differentially expressed metabolites (VIP >1, *p* ≤ 0.05) were identified to contribute to the separation of the two groups in OPLS-DA plot (Figure 2). Among these metabolites, 29 were significantly upregulated post-processing, approximately 34% of which were flavonoids, including acetyl wistin, bonannione A, and hesperidin (Supplementary Table S4). Although the total formononetin content in JD HR did not change significantly after heat processing, two formononetin derivatives, 8-methoxy acetyl ononin and formononetin acetyl glucoside, were significantly upregulated. These results indicated that heat processing significantly changed the HR metabolome and particularly increased the contents of flavonoid derivatives.

### 3.3 Metabolite-metabolite correlation analysis

To reveal the metabolite regulatory network in HR, we performed correlation analysis among the 1,292 detected HR metabolites. The resulted heatmap included 833,986 correlations ranging from −0.9939 to 1 (Figure 2C). When setting the threshold of r^2^ ≥ 0.49 (r ≥ 0.7 or r ≤ −0.7) and *p* ≤ 0.05, 88,955 significant correlations remained including 67,457 positive and 21,498 negative correlations. Flavonoids dominated these significant metabolite-metabolite correlations, with 41,174 significant correlations among 380 flavonoids (32,739 positive and 8,435 negative). Next to flavonoids, there were 30,908 significant correlations among 244 phenolic acids, 20,583 significant correlations among 141 lignans and coumarins, 18,625 significant correlations among 159 terpenoids, and 18,520 significant correlations among 159 alkaloids. When screening highly significant correlations with formononetin by r^2^ ≥ 0.81, 26 correlations were identified (Figure 2D). Four of these 26 correlations were negative, including three alkaloids (hypaphorine, valerine, salicylamide) and one terpenoid (6′-O-Glucosylaucubin). Formononetin had high positive correlation with ten flavonoids, including prunetin, afrormosin, biochanin A, and genkwanin. The other 12 high correlations with formononetin were five lignans and coumarins, four phenolic acids, and three quinones.

### 3.4 Identification of formononetin and CRC-associated targets via network analysis

**FIGURE 3:**
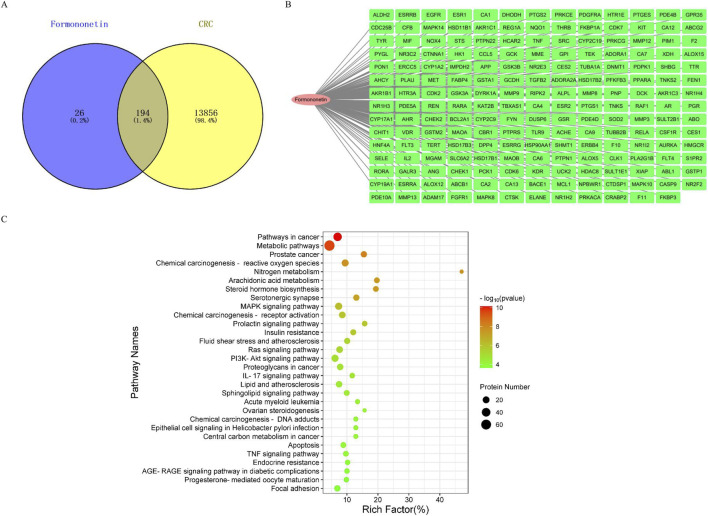
**(A)** Screening of overlapping targets between formononetin and CRC. **(B)** Details of the 194 overlapping targets in the network analysis. **(C)** KEGG pathway enrichment analysis of the 194 targets.

Since it has been established via Zinc database analysis (https://zinc.docking.org/) that formononetin is not a pan-assay interference compound (Bolz et al., 2021), we performed network analysis to search formononetin’s putative molecular targets against CRC. We retrieved 220 predicted targets from SwissTargetPrediction, TargetNet, and PharmMapper databases. A total of 14,050 CRC-related genes were obtained from GeneCards, OMIM, and DisGeNET. The Venn diagram revealed 194 overlapping genes between predicted formononetin targets and CRC-related genes (Figures 3A,B). GO enrichment analysis of the 194 genes identified 517 enriched terms: 358 biological processes (BP), 43 cellular components (CC), and 116 molecular functions (MF). The top 10 enriched terms in each category were shown in Supplementary Figure S1, highlighting key processes such as peptidyl-tyrosine phosphorylation, cytosol localization, receptor complex formation, cytoplasmic signaling, steroid binding, zinc ion binding, and protein kinase activity. KEGG pathway enrichment analysis of the 194 genes identified 114 significantly enriched pathways. The top 30 pathways (Figure 3C) included several cancer-related signaling pathways such as pathways in cancer, MAPK signaling, Ras signaling, and PI3K-Akt signaling. These results suggested that formononetin may exert anti-CRC effects by modulating multiple oncogenic signaling pathways.

### 3.5 Proteomic changes in colon cancer cells treated with formononetin

**FIGURE 4:**
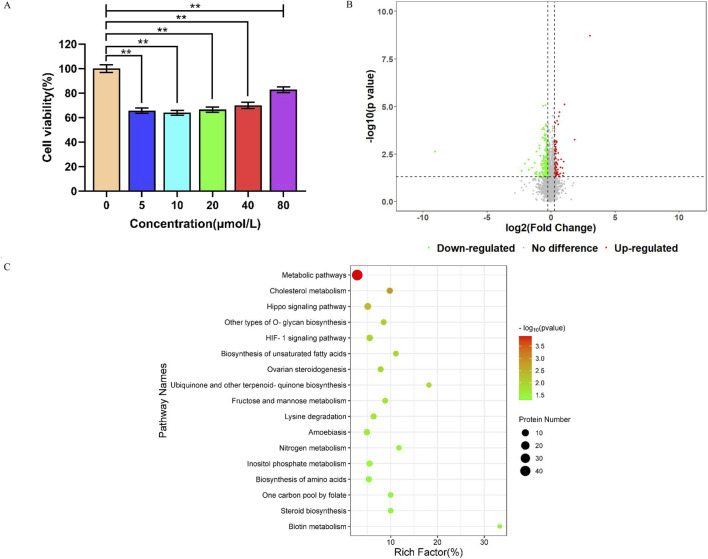
**(A)** Viability of SW620 cells significantly inhibited by different concentrations of formononetin solution at 48 h **p < 0.01. **(B)** Volcano plot generated from the proteomic data of formononetin-treated SW620 cells and controls. **(C)** Pathway enrichment analysis of 291 differentially expressed proteins in formononetin-treated SW620 cells.

We evaluated the inhibitory effect of formononetin on SW620 colon cancer cells by measuring cell viability using the CCK-8 assay. Compared to the untreated control cells, formononetin treatment significantly reduced SW620 cell viability at tested concentrations (5, 10, 20, 40, and 80 μM), although the effect was not strictly dose-dependent (Figure 4A). The concentration of 10 μM was selected for further proteomics study since it gave pronounced inhibitory effect without inflicting overwhelming cytotoxicity. DIA proteomics identified 7,381 proteins, of which 291 were significantly altered upon formononetin treatment, and the fold changes ranged from 0.0019 to 8.2382 (Figure 4B; Supplementary Table S5). Specifically, 105 proteins were significantly upregulated, while 186 proteins were significantly downregulated including CCDC18, CA9, IGF1R, and MME.

GO enrichment analysis of these 291 differentially expressed proteins identified 3,457 enriched BP terms, 372 CC terms, and 404 MF terms. The top ten enriched GO terms in each category were displayed in Supplementary Figure S2, including single-organism cellular process, multicellular organismal process, cellular anatomical entity, cytoplasmic part, protein binding, and carboxylic acid binding. KEGG pathway enrichment analysis revealed 17 significantly enriched pathways, including metabolic pathways, Hippo signaling, HIF-1 signaling, nitrogen metabolism, one-carbon pool by folate, and cholesterol metabolism (Figure 4C). KEGG pathway enrichment analyses of upregulated and downregulated proteins were also conducted and shown in Supplementary Tables S6, S7. These findings indicated that formononetin caused broad proteomic changes involving multiple metabolic and oncogenic pathways in SW620 cells.

### 3.6 Phosphoproteomic alterations in colon cancer cells treated with formononetin

**FIGURE 5:**
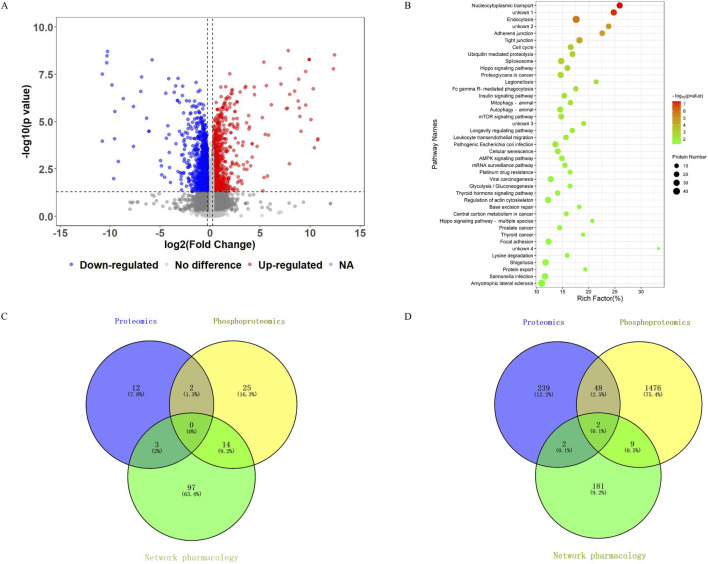
**(A)** Volcano plot generated from the phosphoproteomic data of formononetin-treated SW620 cells and controls. **(B)** Pathway enrichment analysis of 1,535 significantly altered phosphorylated proteins in formononetin-treated SW620 cells. **(C)** Comparison of significantly enriched pathways common among network, proteomics, and phosphoproteomics analyses. **(D)** Comparison of common proteins (targets) generated from network, proteomics, and phosphoproteomics analyses.

Further investigation into formononetin’s molecular mechanisms of cancer cell inhibition was conducted using DIA-based phosphoproteomic analysis. We quantified 19,038 phosphorylation sites, among which 2,587 sites in 1,535 phosphorylated proteins were significantly altered (Figure 5A; Supplementary Table S8). These phosphorylation sites comprised 79.1% phosphoserine, 16.4% phosphothreonine, and 4.5% phosphotyrosine modifications. Among them, 1,244 phosphorylation sites were significantly upregulated (involving proteins such as VAPA, CA9, YY1, FOXK2, SQSTM1), while 1,343 sites were downregulated (in proteins such as AKT2, YWHAB, MME, YAP1). GO enrichment analysis of the phosphoproteins identified 7,832 significantly enriched terms (p < 0.05): 6,104 BP, 898 MF, and 830 CC. The most enriched terms were cellular component organization or biogenesis, protein binding, poly(A) RNA binding, intracellular organelle part, and nuclear compartmentalization (Supplementary Figure S3).

KEGG pathway enrichment analysis highlighted 41 significantly enriched pathways (*p* < 0.05), such as cell cycle, autophagy, Hippo signaling, mTOR signaling, AMPK signaling, glycolysis/gluconeogenesis, lysine degradation, and insulin signaling (Figure 5B). While there was no commonly enriched pathways across network analysis, proteomics, and phosphoproteomics, 19 pathways were enriched by at least two analyses, including Hippo signaling and lysine degradation by both proteomics and phosphoproteomics, and proteoglycans in cancer and glycolysis/gluconeogenesis by both network analysis and phosphoproteomics (Figure 5C). These findings highlighted formononetin’s multifaceted regulatory effects on key oncogenic and metabolic pathways.

### 3.7 Target verification via molecular docking

To further validate key molecular targets of formononetin, we compared proteins identified by network analysis, proteomics, and phosphoproteomics (Figure 5D). Two targets, CA9 and MME, were consistently identified across all three analyses, suggesting their central roles in formononetin-mediated anti-cancer effects. Additionally, 50 proteins were common between proteomic and phosphoproteomic datasets (Supplementary Table S9), while 11 targets, including FYN and DNMT1, were shared between network analysis and phosphoproteomics. We confirmed the binding affinity of formononetin to CA9, MME, and YAP1 (a key regulator of Hippo signaling) by performing molecular docking. Control drugs 5-Fluorouracil, Capecitabine, Oxaliplatin, and Irinotecan were included for comparison. The binding energy scores for formononetin-CA9, formononetin-MME, and formononetin-YAP1 in molecular docking analysis were −4.8162, −6.1931, and −4.3183 kcal/mol, respectively. These scores were higher than those for Capecitabine and Irinotecan, but lower than those for 5-Fluorouracil and Oxaliplatin (Supplementary Table S10). The complete docking analysis results demonstrated that formononetin had strong binding affinity to CA9, MME, and YAP1, indicating they were potential formononetin targets (Supplementary Figure S4).

## 4 Discussion

*Hedysari Radix* (HR) is well-known as a TCM for its diverse pharmacological properties, including antioxidant, anti-tumor, anti-inflammatory, lipid-lowering, and antihypertensive activities. Similar to another traditional Chinese medicine *Astragalus membranaceus* (Fisch.) Bunge [Fabaceae], HR has been traditionally used for its tonic effects on “*Qi*” (Bai et al., 2020; Tsai et al., 2022). Comprehensive understandings of specialized secondary metabolites in TCMs are crucial in understanding TCM’s pharmacological efficacy, and the secondary metabolite profile in HR has not been completely investigated. In this study, we applied targeted UPLC-ESI-QTRAP-MS/MS analysis to profile secondary metabolites in eight heat-processed HR products. A total of 1,292 secondary metabolites including flavonoids, phenolic acids, alkaloids, and terpenoids were identified. Compared to previous studies (Bai et al., 2020; Tsai et al., 2022), our findings expanded the inventory of secondary metabolome of HR, demonstrating the power of metabolomic technologies in elucidating TCM’s molecular composition.

Among the identified metabolites, 380 belonged to the flavonoid group, which were well-documented for their anti-cancer properties in clinical and preclinical studies (Khan et al., 2021). Several flavonoids with known anti-tumor activities, including formononetin, prunetin, kaempferide, medicarpin, and afrormosin, were detected in HR (Wu et al., 2017; Li et al., 2020; Khan et al., 2021; Köksal Karayildirim et al., 2021; Kim et al., 2022; Chen et al., 2023), and each of them were more than 0.5% of the total metabolite content. Besides flavonoids, abundant phenolic acid metabolites such as ferulic acid, isoferulic acid, and acetyl resveratrol were also identified and had reported anti-cancer effects (Tino et al., 2016; Gao et al., 2018; Long et al., 2020). Further validation using TCMSP identified 11 predicted metabolites with potential activity in HR, and six of them including formononetin, naringenin, medicarpin, quercetin, were consistently detected in different HR products.

Heat-processing (pronounced *Paozhi* in Chinese) is an ancient traditional Chinese medicine processing technique to enhance therapeutic efficacy and safety of TCMs. We investigated metabolic variations between unprocessed HR (SJD HR) and other heat-processed HR products including JD HR. The PCA plot of metabolomic data revealed that heat-processing improved HR homogeneity in different HR materials. Moreover, the levels of several pharmacologically important metabolites, such as coumarin, vicenin-3, and rhamnetin-3-O-rutinoside-5-O-rhamnoside, were significantly elevated after heat-processing (Liu et al., 2023; Taherkhani et al., 2023; Todorov et al., 2023). Approximately 8% of the detected metabolites were significantly altered (*p* ≤ 0.05) between SJD HR and JD HR. Notably, 34% of the significantly increased metabolites post-processing were flavonoids, including two formononetin derivatives. So heat processing seemed to enhance HR’s pharmacological potency by increasing levels of flavonoids such as formononetin derivatives. These findings support the long-standing hypothesis that heat-processing could enhance TCM’s efficacy from a molecular basis.

Metabolite-metabolite correlation analysis has been widely used to elucidate key regulatory pathways (Toubiana et al., 2012; Ji et al., 2017), so we also conducted a comprehensive metabolite-metabolite correlation analysis of HR secondary metabolites. A large number of significant correlations were revealed, and over 75% of them were positive correlations. Flavonoids dominated the significant metabolite-metabolite correlations, followed by phenolic acids, lignans and coumarins, terpenoids, and alkaloids. Most of flavonoid-related correlations were positive, while the majority of negative correlations were associated with terpenoids or alkaloids. One of the significant correlations involving flavonoids were between formononetin and biochanin A. These two metabolites have been identified as the most abundant flavonoids in *M. truncatula* roots, and they share structural similarities and both have hepatoprotective and anti-ulcer activities (Sharma and Kabra, 2025). Formononetin is the precursor of afrormosin, and significant correlation between them were also identified (Al-Ani and Dewick, 1980).

Formononetin is notable for its antitumorigenic properties, which have been demonstrated by both *in vitro* and *in vivo* studies. The antitumorigenic properties were reported to function through regulation of multiple oncogenic signaling pathways, such as PI3K/AKT, MAPK, and apoptotic regulators Bax, Bcl-2, and caspase-3 (Tay et al., 2019; Aliya et al., 2023). Recent studies have shown that formononetin could ameliorate DSS-induced colitis by inhibiting MAPK/PPAR-γ/NF-κB/ROS signaling and suppress colitis-associated colon carcinogenesis through regulating lipid metabolism and inhibiting the mTORC2/Akt axis (Cao et al., 2025; Liu et al., 2025). The molecular mechanisms underlying formononetin’s anti-CRC activity, however, remain largely unexplored. In the current study, we conducted an in-depth mechanistic investigation of formononetin’s effects on colon cancer cells by integrating network analysis, proteomics, and phosphoproteomics. Our findings revealed that a broad range of proteins were affected by formononetin treatment to inhibit SW620 colorectal cancer cell’s proliferation. Specifically, 50 proteins and their corresponding phosphorylation sites were identified as key participants in formononetin-mediated anti-tumor activity. Among these proteins, MME, CDH1, APC, and CA9 have not been previously reported to be involved in anti-cancer regulations. MME and CA9 are particularly important since their regulation by formononetin were consistently observed across network analysis, proteomics, and phosphoproteomics.

CA9 is typically expressed at low levels in normal tissues (primarily gastrointestinal tract) but highly upregulated in multiple cancers including CRC (Ronca and Supuran, 2024). Its overexpression correlates with poor prognosis and chemoradiotherapy resistance in CRC patients, making it a potentially important therapeutic target (Kivela et al., 2001; Guedj et al., 2011). The proteomic data revealed that the level of CA9 protein in SW620 cells was downregulated while its phosphorylation at Ser448 was significantly increased following formononetin treatment. Given that dephosphorylation at Ser448 is required for full enzymatic activity, these findings suggested that formononetin may exert anti-cancer effects by disrupting CA9 enzyme function (Ditte et al., 2011). Similarly, MME has also been implicated in tumorigenesis of various cancers including CRC (Haase et al., 2017). Both MME protein levels and its phosphorylation were significantly altered following formononetin treatment in SW620 cells. Molecular docking analysis confirmed that formononetin could strongly interact with CA9 and MME, indicating their critical roles in formononetin’s suppression of colon cancer cells.

Several studies have demonstrated that formononetin could inhibit colon carcinoma cell growth *in vitro* and *in vivo* (Huang et al., 2015; Wang et al., 2018). Our previous study showed that the Yiqi Sanjie (YQSJ) formula, which contained formononetin, was effective in treating stage III CRC patients and AOM/DSS-induced CRC model mice (Wan et al., 2022; Zheng et al., 2022). Several cancer-related pathways regulated by YQSJ were also significantly enriched in the current study, such as cholesterol metabolism and Hippo signaling. The Hippo signaling pathway was significantly enriched in both proteomics and phosphoproteomics analyses. This pathway is highly conserved and pivotal in tumorigenesis, organ size control, and cell proliferation, making it an attractive cancer therapy target (Jin et al., 2023). While the protein levels of YAP1, the central effector of Hippo signaling pathway, did not change following formononetin treatment, its Thr110 phosphorylation was significantly reduced. Since YAP1 phosphorylation is critical for its nuclear localization and transcription, the decreased Thr110 phosphorylation suggested that formononetin might inhibit oncogenic signaling through affecting YAP1’s phosphorylation (Piccolo et al., 2014; Lin et al., 2023). Besides Hippo signaling, 14 other pathways were also significantly enriched in both network and phosphoproteomics analyses, including cancer-associated pathways such as insulin signaling and cell cycle regulation (Aliya et al., 2023). Cholesterol metabolism and HIF-1 signaling pathways in CRC cancer cells were also affected by formononetin treatment, and HIF-1 signaling is closely linked to CA9 function and hypoxic adaptation in tumors. These results highlighted formononetin’s effects in disrupting multiple oncogenic pathways and its potential as a valid cancer therapeutic agent.

To our knowledge, this is the first comprehensive study integrating proteomics and phosphoproteomics to systematically elucidate HR and formononetin’s anti-CRC mechanisms, and potential formononetin targets CA9 and MME were identified. There were several limitations of this study. First, network pharmacology and molecular docking analyses were computational and required further experimental confirmation. These evidences of formononetin’s target engagement were preliminary and not direct. Future investigations should be conducted to validate these predictions according to established pharmacological standards such as the “four pillars of best practice” (Heinrich et al., 2022). Secondly, mechanistic analyses were limited to a single CRC cell line (SW620), potentially restricting generalizability of the results. The response of SW620 cells to formononetin was not strictly dose-dependent, which may stem from cell line-specific sensitivity or biphasic (hormetic) effect, highlighting the need for additional experiments using more cell lines (e.g., SW1116, HCT116) across extended time points (e.g., 24 and 72 h) and wider treatment concentration range (e.g., 1–5 μmol/L) (Jodynis-Liebert and Kujawska, 2020). Finally, the physiological relevance of identified formononetin targets and related pathways need to be validated *in vivo* such as in animal models, which is essential to substantiate formononetin’s therapeutic potential in CRC.

## 5 Conclusion

In summary, this study provided a comprehensive metabolomic characterization of HR, revealed metabolic changes in HR after heat processing. The anti-tumor mechanisms of formononetin in HR were explored, and its potential regulation of multiple targets and signaling pathways were revealed. In particular, regulation of HIF-1-CA9 and Hippo-YAP1 signaling pathways were highlighted as the key mechanisms of formononetin’s anti-CRC activity. These findings offer new insights into the secondary metabolites in HR and the anti-tumor properties of formononetin, provide a foundation for developing novel therapeutic strategies for CRC.

## Data Availability

The datasets presented in this study can be found in online repositories. The names of the repository/repositories and accession number(s) can be found in the article/Supplementary Material.
